# Mircrining the injured heart with stem cell-derived exosomes: an emerging strategy of cell-free therapy

**DOI:** 10.1186/s13287-019-1548-7

**Published:** 2020-01-09

**Authors:** Khawaja Husnain Haider, Beatrice Aramini

**Affiliations:** 1 Sulaiman Alrajhi University, Al-Qaseem, Kingdom of Saudi Arabia; 2 Division of Thoracic Surgery, Department of Medical and Surgical Sciences, University of Modena and Reggio Emilia, Modena, Italy; 3 Department of Basic Sciences, Sulaiman Alrajhi University, PO Box 777, Al Bukairiyah, 51941 Kingdom of Saudi Arabia

**Keywords:** Bone marrow, Exosomes, Mesenchymal, Microvesicles, MSCs, myocardial, paracrine

## Abstract

Bone marrow-derived mesenchymal stem cells (MSCs) have successfully progressed to phase III clinical trials successive to an intensive in vitro and pre-clinical assessment in experimental animal models of ischemic myocardial injury. With scanty evidence regarding their cardiogenic differentiation in the recipient patients’ hearts post-engraftment, paracrine secretion of bioactive molecules is being accepted as the most probable underlying mechanism to interpret the beneficial effects of cell therapy. Secretion of small non-coding microRNA (miR) constitutes an integral part of the paracrine activity of stem cells, and there is emerging interest in miRs’ delivery to the heart as part of cell-free therapy to exploit their integral role in various cellular processes. MSCs also release membrane vesicles of diverse sizes loaded with a wide array of miRs as part of their paracrine secretions primarily for intercellular communication and to shuttle genetic material. Exosomes can also be loaded with miRs of interest for delivery to the organs of interest including the heart, and hence, exosome-based cell-free therapy is being assessed for cell-free therapy as an alternative to cell-based therapy. This review of literature provides an update on cell-free therapy with primary focus on exosomes derived from BM-derived MSCs for myocardial repair.

## Background

Nearly two decades of bone marrow stem cell (BMSC) research for the treatment of the infarcted heart has generated encouraging data in experimental animal models as well as in the clinical settings and shown the safety and effectiveness of the procedure as an alternative to the contemporary therapeutic modalities [1]. The transplanted BMSCs attenuate infarct size expansion, prevent left ventricular remodeling, and preserve the indices of global cardiac function [2]. The much-purported mechanism that the transplanted BMSCs cross lineage-restriction and adopt morphofunctionally competent cardiomyocyte phenotype for de novo myocardial regeneration has been challenged by various research groups [3, 4]. Amid the controversy regarding the differentiation capacity of the transplanted BMSC, paracrine release of bioactive molecules has emerged as an alternative and more acceptable mechanism by which stem cells contribute towards preserved global cardiac function post-transplantation in the infarcted myocardium [5, 6]. Nevertheless, the paracrine secretome of BMSCs does not possess a distinct composition; the amount, as well as the composition of the paracrine secretome of BMSC, is influenced by a multitude of factors encompassing the microenvironment in which the cells are present to the physical, pharmacological, or genetic manipulation of the cells [7]. We have also reported that preconditioned mesenchymal stem cells (MSCs) and MSCs genetically modified to overexpress microRNA-210 (miR-210) transferred miR-210 to the juxtaposed cardiomyocytes in a direct co-culture system in vitro as well as to the recipient cardiomyocytes post-transplantation. Our results vividly showed that the transfer of miR-210 occurred from the transplanted MSCs to cardiomyocyte *via* gap junctions [8, 9]. We also observed that the transferred miR-210 initiated survival signaling in the recipient cardiomyocytes and contributed to their survival upon subsequent exposure to lethal anoxia.

Encouraging results from the use of paracrine secretions of stem cells in general and from the BM-derived MSCs, in particular, have paved the way for cell-free therapy which is based on the engineering of cells to tailor their secretions to the therapeutic needs [10, 11].

**Fig. 1:**
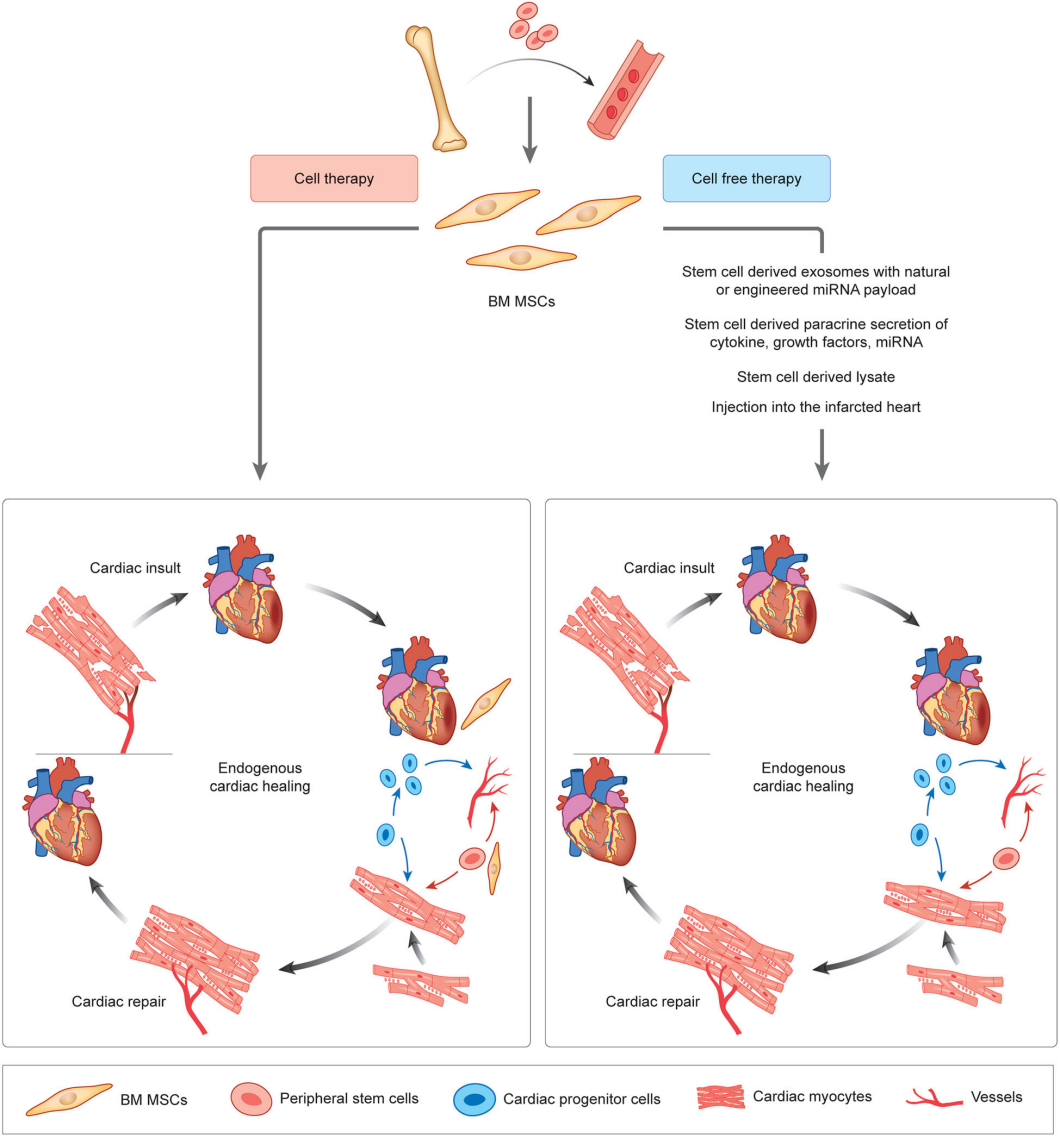
A head-to-head comparison of cell therapy and cell-free therapy

Figure 1 shows a head-to-head comparison of cell therapy and cell-free therapy. A step forward in this regard is the use of stem cell-derived exosomes, either with their intrinsic miR payload or with the manipulated miR payload of interest for which they are used as carriers for myocardial delivery [12]. Our review summarizes the advancements in this fast-emerging therapeutic strategy with immense therapeutic potential and provides a critical appreciation of its various aspects in the clinical perspective.

## BM-derived MSCs

Bone marrow (BM)-derived mesenchymal stem cells (MSCs) are one of the most well-characterized and extensively studied cell types in the field of stem cell-based therapy. They are a heterogeneous group of cells that constitute an integral part of the stem cell niche in the BM and also support the hematopoietic stem cell (HSC) niche microenvironment by secreting a plethora of growth factors and cytokines to regulate their activity [13, 14]. Given the lack of a consensus marker for identification, they are generally characterized based on their plastic adherence properties; tri-lineage differentiation potential, i.e., osteogenic, adipogenic, and chondrogenic; and surface membrane expression of specific clusters of differentiation (CD) including CD90 and CD105, besides CD17, CD29, CD44, and CD106, while lacking in the expression of HSC-specific markers, i.e., CD31, CD34, CD133, CD14, CD19, and KDR. This is in line with the recommendations of International Society for Cell Therapy and Gene Therapy (ISCT) to establish uniform criteria for isolation and purification of MSCs for therapeutic application [15, 16]. MSCs are available from almost every tissue, i.e., adipose tissue, umbilical cord, dental pulp, etc., but their isolation from the BM is most favored due to the ease of accessibility and the requirement of less invasive protocols. The percentage of isolated MSCs from the BM is 0.001–0.0001% only; however, they can be easily expanded in vitro to obtain a larger number. It is important to note that MSCs isolated from various species and various tissues may diverge in the expression of surface markers. They have been studied in-depth for reparability of the heart besides other clinical applications due to near-ideal characteristics, i.e., ease of autologous availability and undifferentiated in vitro expansion, multi-lineage differentiation potential, immunomodulatory characteristics, and multifactorial mechanisms of myocardial repair including the release of bioactive molecules as part of their paracrine action [17].

## MSCs and their paracrine activity

BM-derived MSCs release a variety of bioactive molecules for intracellular communication and signaling in their vicinity. The paracrine hypothesis was earlier proposed for the interpretation of the therapeutic benefits of stem cell therapy. According to the paracrine hypothesis, akin to any other cell in the body, stem cells actively secrete many different substances, i.e., chemokines, cytokines, interleukins, growth factors, lipids, steroids, nucleotides and nucleic acids, ions, metabolites, etc. Moreover, the release of microRNAs (miRs) is an integral part of their paracrine activity which will be discussed further as part of exosomal activity [18]. These substances are secreted either by conventional or by unconventional pathways in a regulated or unregulated manner [19]. The composition of secretome (consisting of the mixture of soluble factors as well as microvesicles) released by each stem cell type is unique and cell-specific in composition. The composition of the secretome is also affected by a multitude of extraneous factors including the stem cell donor-specific factors, i.e., age, health status, comorbidities, tissue source, etc., as these factors significantly impact the quality of the donor stem cells as well. It is pertinent to mention that the in vivo spectrum of bioactive molecules released by stem cells remains oblivious due to the lack of a standard protocol. Single nucleus capture microdissection combined with high-throughput real-time polymerase chain reaction (PCR) was performed to elucidate gene expression profile of the transplanted MSCs in an infarcted mouse heart model of myocardial infarction (MI) which revealed that the expression profile was comparable with the cells cultured in vitro [20]. The transplanted cells were rich in VEGF, IGF-1, FGF-2, Ang-1, Ang-2, PDGF, NGF, BMP-2 & 4, IL6, TGF, and TNF expression in the infarcted hearts as compared to the normal hearts. This observation also signified the role of microenvironment as an important determinant of the paracrine expression profile of a cell. A similar earlier study has shown heterogeneity in the paracrine profile shown by the different lineages of BM-derived cells wherein single nucleus PCR revealed higher-level expression of angiogenic growth factors by CD45+ subpopulation as compared to the other sublineages [21].

In vitro profiling of stem cell secretome mostly relies on the conditioned medium obtained from the cultured cells. Despite extensive efforts, a standard secretome profile in vitro is lacking that could be used as a reference as the composition of the secretome fluctuates in response to multitude of factors including the type of the stem cell, cell source, its differentiation status, and microenvironmental cues which are duly responded by the cells to maintain cellular homeostasis. For example, the secretome of naïve undifferentiated BM-derived MSCs is richer in pro-angiogenic factors as compared to the secretome of their osteogenic and chondrogenic derivatives [22]. Similarly, the secretome of MSCs subjected to ischemia varied in VEGF expression between the cells isolated from C57/BL6 and Balb/c mouse strains [23]. This difference was also evident from the angiogenic response observed when the cells from the two sources were engrafted in an experimental model of hind-limb ischemia. The authors attributed the difference between the angiogenic reparability of the cells and the genetic makeup of the donor animals used as a cell source, i.e., polymorphism in the *cis*-acting VEGF gene in Balb/c mice on chromosome 17 significantly reduces VEGF gene transcription as well as expression under ischemic conditions. A recently published study has reported that MSCs subjected to hypoxia were richer in pro-inflammatory and pro-angiogenic cytokine expression (especially in VEGF-A expression) as compared to their counterparts cultured under normoxia [24]. MSC secretome profile changes drastically in 3D culture as compared to the 2D culture conditions as the former mimics more closely to their natural habitat [25, 26]. The secretome profile changes thus observed have been attributed to the expression of the desirable phenotype of the cells due to spheroid formation thus rendering a more desired physiological microenvironment. Priming 3D-cultured human MSCs with interleukin-1 enhanced the paracrine release of GCSF, VEGF, and interleukin-1 receptor antagonist (IL-1Ra). Protein array showed a more potent immune profile which was required to orchestrate an effective tissue repair [27]. The effect of culture surface topology and microenvironment significantly alters the morphology of the cultured cells besides altering their cellular activity including the secretome profile [28]. The authors used a high-throughput tool TopoWell Plate with unique topographies to quantify the effect of surface topology in terms of cytokine and growth factor release. The results of the study showed a significant relationship between cytokine and growth factor secretion and the cell shape adaptations.

Various strategies are being developed to prime/precondition the cells such that their secretome can be manipulated to achieve the desired composition for cell-free therapy. These strategies include physical manipulation of cells, i.e., ischemic preconditioning [29–32], hypoxic preconditioning, heat-shock treatment [33–35], electrical treatment and mechanical stimulation [36, 37], shockwave treatment [38, 39], and mechanical stress [40]; pharmacological treatment of cells with preconditioning agents, i.e., diazoxide, statins, and PDE5 [41–44]; pre-treatment of cells with growth factors and cytokines, i.e., IGF-I, SDF-1, TGF-b, and IL-1 [45–49]; treatment with cell lysate [50]; or combined treatment using more than one of these strategies [51]. Besides, genetic manipulation via single or multiple gene modification of cells encoding for growth factors, cytokines, pro-survival molecules, or a combination of growth factors with pro-survival factors has been extensively studied to modulate stem cell’s paracrine behavior [52–58]. While elucidating the mechanism underlying the improved paracrine activity of MSCs with concomitant overexpression of Akt and Ang-1, we observed significant induction of HIF-1α and its dependent array of angiogenic growth factors [59]. Such manipulation of the cells not only enhanced their paracrine activity but also altered their culture characteristics as well as resulted in stable therapeutic benefit post-engraftment in the experimental animal models of myocardial infarction [60, 61]. The paracrine factors released from the preconditioned cells initiate diverse signaling pathways. Besides contributing to cytoprotection of the recipient cardiomyocytes, they also support the survival of the transplanted stem cells, initiate an endogenous angiogenic response, and create a favorable concentration gradient of the secreted growth factors to promote endogenous stem cell mobilization, homing-in, and retention in the infarcted myocardium to participate in the repair process [62, 63].

## Exosomes as part of paracrine activity

Exosomes are one of the many subtypes of lipid membrane nano-sized vesicles ranging from 50 to 200 nm in size and secreted by various cell types for intercellular cross-talk [64, 65]. Characterized by the lipid bilayer structure, exosomes are quite distinct from microvesicles that are much larger in size while exosomes differ from apoptotic bodies which are derived from apoptotic cells and contain nuclear fragments [66]. Proteomic analysis showed that MSC-derived exosomes retained critical surface markers, receptors, and functional proteins akin to their mother cells which provided a comprehensive understanding of the mechanism by which MSC-derived exosomes contributed towards tissue repair and regeneration [67]. There was heterogeneity in the exosomes released by cells in terms of their size as well as contents which have reported the existence of distinct subpopulations of exosomes [68]. The exosome subpopulations, categorized as low-density (LD) and high-density (HD) exosomes, were observed during sucrose density gradient centrifugation. Nevertheless, exosome subpopulations expressed exosome-specific protein markers including tetraspanins, i.e., CD9, CD63, and CD81; biogenesis-related specific marker proteins, i.e., TSG101 and ALIX; and heat-shock proteins (HSP), i.e., HSP60, HSP70, and HSP90. Raman spectral analysis of individual exosomes isolated from a single cell line showed high-level spectral variability in terms of cholesterol, protein, lipid, and cytosolic contents [69]. The authors of this study also compared the analysis of exosomes from eight different cell lines and observed at least four subpopulations of exosomes were conserved across the cell lines thus pointing towards their preserved biological functionality. On a functional basis, exosomes are responsible for signal transduction affecting the physiological and pathological working of cells besides being part of antigen presentation and immune response mechanisms [70]. Exosome heterogeneity may also be attributed to differences in the isolation protocols, their secreting cell source, and methods used for their characterization [71]. Moreover, the efficiency of exosome production has been reported as inversely related to the developmental maturity of the donor of MSCs. In a direct comparison of MSCs derived from ESCs, fetal tissue, and umbilical cord, it was observed that the ESC-derived MSCs were most efficient while umbilical cord-derived MSCs were least efficient in exosome production thus underpinning an inverse relationship between the developmental stage and rate of exosome production [72].

Originating as intraluminal vesicles, exosomes are released extracellularly when an intermediate endocytic compartment in the cell fuses with the plasma membrane and gets extruded into the extracellular *milieu* [73]. Although the exact mechanism underlying their biogenesis remains an area of intense investigation, it is generally considered as an “endosomal sorting complex required for transport” (ESCRT)-dependent or ESCRT-independent mechanism [74]. Besides, ceramide has been implicated in the biogenesis and secretion of exosomes. At molecular levels, Rab proteins, i.e., Rab11, Rab27, and Rab35, are involved in intracellular compartment trafficking and ultimately secretion of exosomes with an as yet unconfirmed role of SNARE proteins [75]. Once released from the cells, exosomes fuse with the recipient cell membrane and get internalized to deliver their payload. Our review is more focused on the exosome-based mircrine activity of MSCs of BM origin with focus on their potential use in cardiovascular applications as part of cell-free therapy which is being extensively studied in the context of regenerative and reparative strategies for the infarcted heart [76, 77].

## Composition of exosome-based mircrine activity of MSCs

The exosomal payload includes a variety of biomolecules such as DNAs, mRNAs, miRs, non-coding RNAs, proteins, lipids, and other cellular metabolites. Since the publication of the first report that exosomes can mediate the transfer of genetic material between two cells [78], many subsequent studies have confirmed that miR transfer between nearby cells (without cell-to-cell contact) may occur via exosomes as part of the mircrine activity of the cells [79–81]. MiRs are small non-coding RNA molecules which are 18–22 nucleotides in length. They are produced as inactive precursors in the nucleus which undergo multiple-step processing that involves enzyme cleavage and subsequent exportation into the cytoplasm. Upon functional maturity, miRs post-transcriptionally regulate gene expression to affect multiple cellular functions including cell survival, proliferation, and differentiation. Nucleic acid content analysis of MSCs’ derived exosomes has revealed the specific presence of both pre-miRs and miRs as an integral part of the exosomal payload [82]. A direct comparison of miR expression profile of rodent BM MSCs and their derivative exosomes revealed a general similarity; however, some miRs which negatively regulate cardiac function, i.e., miR-130, miR-378, and miR-34, while others which positively impact cardiac function, i.e., miR-29 and miR-24, were differentially expressed between them [83]. The similarity between MSCs and their derivative exosomes in terms of miR profile points to the identical mechanism of beneficial effects of their therapeutic applicability. Nevertheless, despite extensive profiling studies, a single standard MSC miR profile is still lacking that can be used as a reference. It keeps changing even between the cells derived from the same tissue but cultured under a different set of culture conditions and differentiation status of the cells as well as passage number. Therefore, the miR profiling of MSCs only reveals the expression of signature miRs by the cells under a specific set of conditions. This is akin to the composition of other paracrine secretions of MSCs which keep changing in response to various extraneous factors in the microenvironment of a cell. This variability in the exosomal payload of allows the cells to respond to the functional requirements of the cells and their response to the pathophysiological cues. For example, miR-572 and miR-638 may be used to distinguish between the early and late passage MSCs in vitro [84]. Similarly, global miR profiling showed 15 miRs showing high-level changes, with miR-222 and miR-423 showing the most significant contribution during osteogenic differentiation of MSCs [85]. A direct comparison of the three cell populations profiled at different osteogenic differentiation states, i.e., naïve, un-manipulated early-stage, and late-stage osteogenic cells, showed that from amongst the top 50 miRs expressed in these cells, 42 miRs (84%) showed similar expression levels [86]. From amongst the differentially expressed miRs, the expression of the negative regulators of osteogenesis including miR-31, miR-144, and miR-221 was significantly decreased in the exosomes derived from the late-stage differentiating MSCs. A similar study revealed differential expression of miRs including let-7a, miR-199b, miR-218, miR-148a, miR-135b, miR-203, miR-219, miR-299-5p, and miR-302b which increased while miR-221, miR-155, miR-885-5p, miR-181a, and miR-320c decreased significantly in exosomes derived from MSCs undergoing osteogenic differentiation until 7 days of observation as compared to the undifferentiated cells [87]. Microarray analysis also revealed a series of upregulated miRs, i.e., miR-193a-5p, miR-320c, and miR-92a, in the exosomes derived from human BM MSCs undergoing chondrogenic differentiation [88].

Akin to differentiation status, tissue source of MSCs has significant bearing on exosome release and their miR contents [89]. Although the authors of the study found significant similarity in terms of miR contents of the exosomes derived from adipose tissue and BM-derived MSCs, the relative proportion of the most representative miRs was different between MSCs derived from the two distinct tissue types. This difference was attributed to the cues emanating from their respective microenvironment. Similar differences have been observed in the exosomes isolated from embryonic stem cell (ESC)-derived MSCs and adult tissue-derived MSCs [90]. On the same note, the health status of the MSC donor remains an important determinant of the miR contents of their derivative exosomes [91, 92].

## Manipulation of MSCs to enhance exosomal miR payload

In addition to the development of Good Manufacturing Practice (GMP) grade protocols to enhance the exosomal activity of MSCs [93], the cells are being manipulated to modify their exosomal miR payload [94]. Various strategies have been adopted in this regard. For example, the treatment of in vitro cultured MSCs with ischemic brain extract leads to miR-133-rich exosome release by the cells [95]. Genetically modified MSCs with an expression plasmid encoding for miR-146 or miR-584 released exosomes rich in their respective miR and were successfully used to treat glioma in experimental rodent and murine animal models [96, 97]. Overexpression of HIF-1α in MSCs not only increased their rate of exosome secretion but also altered the miR payload of the secreted exosomes from the genetically modified MSCs. Profiling of exosomes derived from HIF-1α overexpressing MSCs showed significantly higher presence of miR-15, miR-16, miR-17, miR-31, miR-126, miR-145, miR-221, miR-222, miR-320a, and miR-424 as compared to control MSCs [94]. From amongst the profiled miRs, miR-31 was of most interest in terms of its role in migration and tube formation response during in vitro angiogenic assay.

MSCs have been manipulated in vitro to alter the expression of various myo-miRs (myocardium-related miRs) including miR-1, miR-133, miR-208, and miR-499 to enhance their cardiac differentiation [98–100]. A recent study has explored an interesting aspect of myo-miRs regarding their exosome-encapsulated release from the infarcted heart (except for miR-133 which is partially released in the exosomes) that gets transferred to the BM mononuclear cells [101]. The recipients’ BM cells consequently respond by decreasing CXCR4 expression which promotes their extravasation from the BM into the peripheral circulation to aid in the myocardial repair process. Peinado et al. have previously reported a similar mechanism in which melanoma cells metastasize as well as send signals to the BM progenitor cells to mobilize out from the BM [102]. Similar results have been reported by other research groups specifying evidence of a dynamic exosomal miR transfer between the injured cells and stem cells as an integral part of the repair process. The recipient stem cells in turn either reprogram their phenotype to become part of the injured tissue or release exosome-encapsulated genetic information which facilitates the surviving cells in the injured tissue to re-enter into cell cycle and become part of the repair process [82, 103]. These data support the hypothesis that microvesicles emanating from the injured tissue cells send “SOS” signals to activate stem cells and ensure their participation in the repair process. We have also reported their role as important regulators of the paracrine activity of MSCs [104].

As stated earlier, myo-miRs have been implicated in the early- and late-stage development of the heart as well as during physiological and pathological conditions. For example, myo-miRs show dysregulated expression in MI patients [105] while downregulation of miR-133a/b has been correlated in MI patients with ventricular fibrillation [106]. Hence, the transplantation of MSCs with transgenic miR-133 is cardioprotective for the infarcted heart [107]. MSCs with miR-133 overexpression also show a higher rate of survival under hypoxic culture conditions as compared to their naïve counterparts. Apart from myo-miRs, various other miRs have been studied in this regard. MiR-21 with anti-apoptotic activity gets dysregulated in the exosomes derived from stromal cells in heart failure patients which significantly impair their regenerative capacity [108, 109]. We have reported that MSCs modified for miR-210 overexpression, either by preconditioning or by genetic modification, transferred miR-210 to the adjacent cardiomyocytes in the co-culture as well as post-transplantation in the infarcted heart. We observed that miR-210 was transferred to the cardiomyocytes* via *gap junctions and prevented the recipient cardiomyocytes’ apoptosis under anoxia during co-culture in vitro as well as in the infarcted heart post-engraftment [8, 9].

In continuation of these observations and with the recent emerging interest in exosomes as safe and efficient mediators of material transfer between cells, an exosome-based cell-free therapy approach has gained popular acceptance for miR delivery and manipulation in cardiovascular settings. Furthermore, exosomes have emerged as an integral part of the multifactorial underlying mechanism of the beneficial effects of cell therapy [110]. Incidentally, exosomes derived from various cell types including CSCs and endothelial cells have been used as part of the cell-free therapy [111–116]. Most of the published reports have used exosomes containing either endogenous or exogenous miR payload. For example, treatment with MSC-derived exosomes enhanced myocardial cell viability through activation of PI3/Akt signaling and concomitant increase in ATP thus resulting in attenuated remodeling in the ischemic heart [115]. A recent study has reported that MSC-derived exosomes alter the polarization status of M1 macrophages to M2 type as the underlying mechanism involving miR-182 to attenuate ischemia/ reperfusion myocardial injury [116]. Moreover, treatment with MSC-derived exosomes also promoted the angiogenic response that helped in regional blood flow recovery in the myocardium [117] besides improving the microenvironment in the infarcted heart by attenuating the ongoing inflammatory response [118].

MSCs have also been physically or genetically modulated to augment miR payload in their secreted exosomes thus boosting their therapeutic potential [119, 120]. MSC-derived exosomes are electroporated with the desired miR mimics to enhance miR payload of interest and later used for treatment. Ma and colleagues have reported miR-132-rich exosomes to promote angiogenesis in the murine heart model of MI [121]. In an interesting study, MSC-derived exosomes were used to precondition tissue cultured cardiac stem cells (CSCs) which enhanced their myocardial reparability post-engraftment [122]. In vitro characterization of the preconditioned CSCs showed their proangiogenic ability in a dose-dependent manner. Profiling of miR revealed that a set of miRs was differentially altered in the preconditioned CSCs (17 miRs increased while 5 showed decreased expression) that contributed to their enhanced migration, proliferation, and tube formation in vitro. Zilun et al. genetically modified MSCs using lentiviral vectors encoding for miR-181a and subsequently used the derivative exosomes to treat ischemia-reperfusion injury-induced inflammatory response in murine myocardium [120]. Echocardiography on day 7 after exosome treatment revealed improved ejection fraction and fractional shortening as compared to control animals treated with phosphate-buffered saline. Molecular mechanisms revealed significantly abrogated expression of pro-inflammatory factors including IL-6, IL-10, and TNF.

## Use of exosomes as delivery vehicles for miRs

Extending further, exosomes have been directly modified for their payload for use as carriers of miRs for delivery to the target cells due to their ability to transfect a wide variety of cells. Their potential to transfect various cell types is being equated with viral vectors for their application as nano-size theranostic delivery platforms for miRs [123]. In addition to their privileged transfection efficiency, exosomes are less cytotoxic, low in immunogenicity, and more compatible than the cells from which they have been derived [124]. In this regard, MSCs are being tipped as the ideal cells for mass-scale exosome production for subsequent engineering to deliver miRs of interest [125]. Protocols have been designed to engineer exosomes directly with the payload of miRs by electroporation, lipofection, sonication, and calcium chloride treatment. For example, MSC-derived exosomes have been successfully loaded to carry miR-132 mimics by electroporation. The miR-132-loaded exosomes were then incubated with HUVECs to upregulate miR-132 in the recipient cells which showed higher angiogenic potential during matrigel plug angiogenesis assay in vitro and post-transplantation in a murine model of acute MI [121]. On the same note, plasma-derived exosomes from healthy donors were engineered to carry miR-31 and miR-451b with anti-tumor activity. Subsequent treatment of hepatocellular carcinoma cells with exosomes loaded with miR-31 and miR-451b significantly enhanced their apoptosis by suppressing anti-apoptotic signaling pathways [126]. Given the successful use of exosomes as vehicles for miR payload delivery, exosome mimics are being developed for delivery of miR payload of interest which includes nano-vesicles generated by cell extrusion and the cell membrane cloaked nanoparticles [127].

## Conclusion and future perspective

The strategy of cell-free therapeutic intervention started with the earlier observations that conditioned medium from genetically modified cells led to improved cell survival and protected the infarcted heart [53, 54]. Since then, favorable data in this regard have kept pouring in to show that the secreted bioactive molecules are cytoprotective, anti-apoptotic, pro-proliferative, and effective in preconditioning of stem cells to support their cardiomyogenic differentiation [128–130]. Efforts are underway to develop a standard protocol for bioprocessing and quality control of the conditioned medium for optimal therapeutic usage [131]. The exosome-based strategy is fast-emerging as an alternative to the conditioned medium-based cell-free therapy approach [132]. Either way, the intent of cell-free therapy is to support the intrinsic repair mechanism of the heart rather than treatment with stem cells from exogenous source

**Fig. 2:**
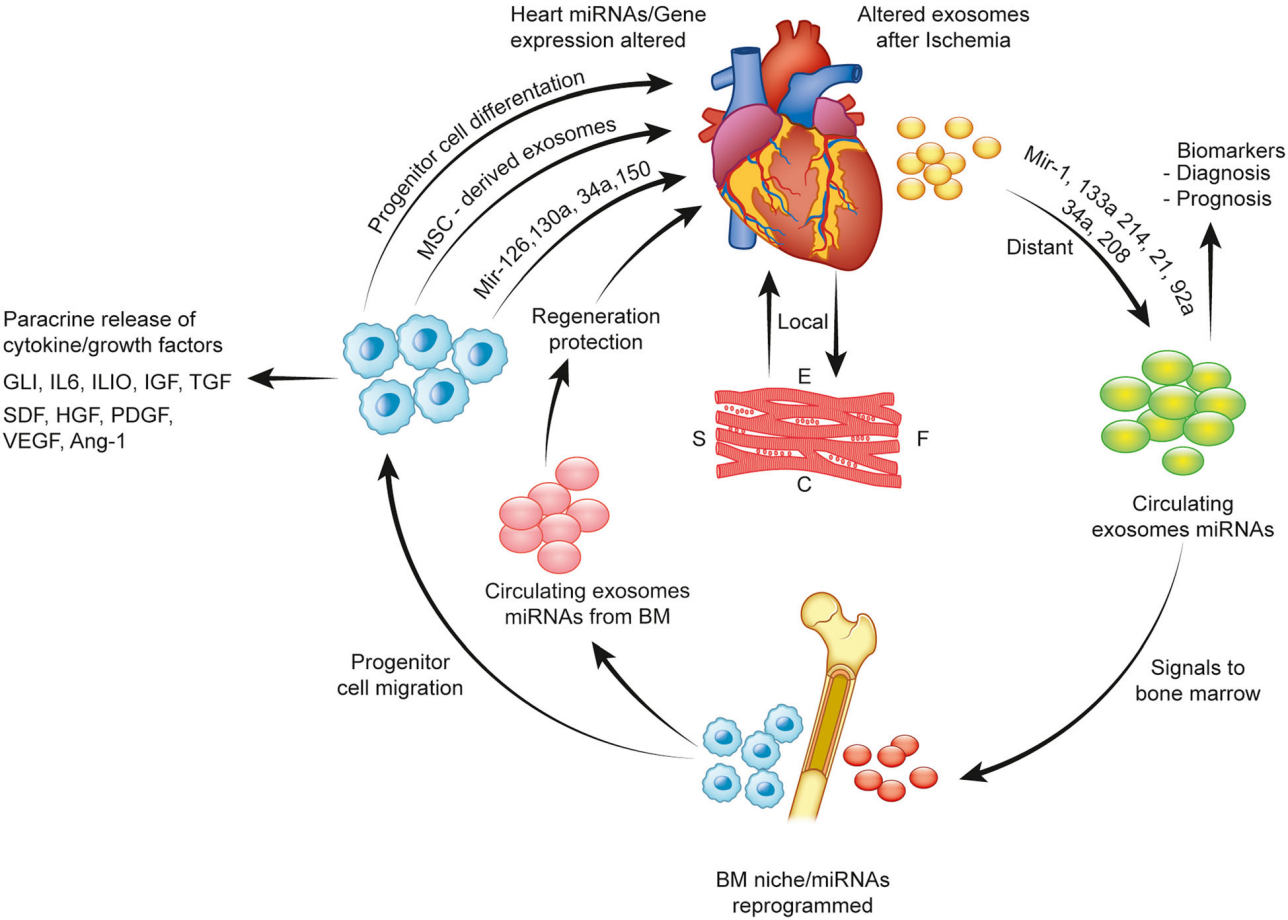
Summary of the role of bone marrow MSC-derived exosomes and miRs in cell-free therapy

(Fig. 2). In addition to the other attributes, cell-free therapy could be rendered logistically more favorable by freeze-drying the secretome for ready-to-use off-the-shelf availability that makes it clinically more relevant [133].

The exosomal payload plays a significant role in the pathophysiology of IHD including endothelial cell function, lipid deposition and plaque formation, and ischemia-reperfusion injury by constituting an integral part of the intercellular communication. These exosomes are released by a diverse population of cells including endothelial cells, cardiomyocytes, and smooth muscle cells which are participating in the disease process as shown in Table

**Table 1:** Exosomal miRs released from various cell types [134, 135]

Cell type	Exosomal miRs released
Endothelial cells	miR-10, miR-143/145, miR-214, miR-342-5p
Smooth muscle cells	miR-155, miR-221/222
Cardiomyocytes	miR-30a, miR-320
Cardiac fibroblasts	miR-27a, miR-28a, 34a

1 [134, 135]. The exosomal miRs released by these cells, as well as by the inflammatory cells homing-in to injured myocardium, also play a significant role in the acute phase inflammatory response as part of the intrinsic repair process in the heart [134]. This necessitates in-depth future studies to develop protocols to ensure that the exogenously delivered exosomes could deliver their miR payload without getting eliminated from the site of injury by the inflammatory cells. It is pertinent to mention that the payload of the released exosomes, including the miR contents, has a dynamic nature, as it keeps changing in response to the microenvironmental factors. Hence, there is as yet no well-defined miR profile available and there are no optimally defined conditions to culture MSCs and reproducibly harvest clinically effective exosomes [136]. Akin to the quality of the donor cells as determinant of the outcome of cell therapy procedure, it is imperative to establish a relationship between the therapeutic effectiveness of exosomes released by various qualities of the cells in culture. Various research groups are currently engaged in optimizing protocols to pack exosomes with therapeutically effective well-defined payload of miRs [137, 138]. A step forward is the use of induced pluripotent stem cells (iPSCs) derived MSCs as a renewable source of exosomes [139]. Another important step forward would be to enhance exosomal tropism for the cardiomyocytes [140]. The expression of the cardiomyocyte-specific peptide on the exosomal surface may improve homing-in of the delivered exosomes and promote their fusion with the recipient cardiomyocyte membrane as a mechanism for the delivery of their miR payload via endocytosis. As in vivo biodistribution of the delivered exosomes is determined by the route of administration [141], future studies would be required to ascertain the effectiveness of various routes of exosome administration in general and intracoronary (I/C) delivery in particular as I/C administration exosomes have been reported as less efficacious in large animal models [142]. On the same note, it is imperative to understand that the acute phase inflammatory response in the infarcted heart, as part of the intrinsic repair process, immensely reduces the efficacy of cell-based therapies as well as exosome-based intervention.

In conclusion, therapeutic benefits of cell therapy are being attributed to the donor cell-derived paracrine factors as well as extracellular vesicles which are loaded with various biologically active components including miRs. As the quality of these secretions of MSCs is amenable to various physical and genetic modulation strategies, future studies should be focused on the bioengineering of cells which should release therapeutically active exosomes with desired miR payload.

## Data Availability

Not applicable.

## Abbreviations

Ang-1: Angiopoietin-1
Ang-2: Angiopoietin-2
BM: Bone marrow
BMP-2 & 4: Bone morphogenetic protein-2 and 4
BMSCs: Bone marrow stem cell
CD: Clusters of differentiation
CSCs: Cardiac stem cells
ESCRT: Endosomal sorting complex required for transport
ESCs: Embryonic stem cells
FGF-2: Fibroblast growth factor-2
HIF-1α: Hypoxia inducible factor-1α
HSP: Heat-shock proteins
IGF-1: Insulin-like growth factor
IL6: Interleukin-6
iPSCs: Induced pluripotent stem cells
ISCT: International Society for Cell and Gene Therapy
miR: MicroRNA
miR-210: MicroRNA-210
MSCs: Mesenchymal stem cells
NGF: Neuronal growth factor
PCR: Polymerase chain reaction
PDGF: Platelet-derived growth factor
TGF: Transforming growth factor
TNF: Tumor necrosis factor
VEGF: Vascular endothelial growth factor
